#  Avian Influenza Virus (H5N1) Replication in Feathers of Domestic Waterfowl

**DOI:** 10.3201/eid1401.071036

**Published:** 2008-01

**Authors:** Yu Yamamoto, Kikuyasu Nakamura, Masatoshi Okamatsu, Manabu Yamada, Masaji Mase

**Affiliations:** * National Institute of Animal Health, Tsukuba, Ibaraki, Japan

**Keywords:** ducks, feathers, geese, Influenza A virus, H5N1 subtype, dispatch

## Abstract

We examined feathers of domestic ducks and geese inoculated with 2 different avian influenza virus (H5N1) genotypes. Together with virus isolation from the skin, the detection of viral antigens and ultrastructural observation of the virions in the feather epidermis raise the possibility of feathers as sources of infection.

Since 1997, an epidemic of avian influenza (AI) virus subtype H5N1 has spread in Asia, causing fatal infections in poultry, wild birds, mammals, and humans (*1*). Wild waterfowl, including ducks and geese, are natural hosts of AI virus of all 16 hemagglutinin subtypes in nature (*2**,**3*). Generally, AI virus is transmitted by the fecal-oral route without causing clinical signs (*2**–**4*). Although current AI virus (H5N1) strains have mild to severe pathogenicity in waterfowl (*5**–**7*), these birds can still be carriers of the virus (*7*). Even asymptomatic domestic ducks can shed the virus from the cloaca and oral cavity (*7**,**8*) and contribute to viral maintenance and spread (*9**,**10*). Therefore, focusing on the epidemiologic role of domestic waterfowl in AI (H5N1) outbreaks is important.

We previously reported that the Japanese AI virus (H5N1) isolated in 2004 causes necrosis of the feather epidermis with viral antigens in domestic ducks, a finding that demonstrates the possibility of viral release from feathers (*11*). In addition, these affected feathers can cause infection in orally inoculated domestic ducks (*12*). Except for our previous studies, to our knowledge this feather lesion has not been reported in AI (H5N1)–infected waterfowl. However, if the feather lesion is common to other waterfowl species and AI (H5N1), affected feathers might involve the spread of the virus. We describe the pathologic, virologic and ultrastructual findings of the feather in domestic waterfowl infected with AI (H5N1).

## The Study

Two species of domestic waterfowl, ducks (n = 4) and geese (n = 4), were used. Domestic ducks (*Anas platyrhynchos* var. *domestica*) called *Aigamo* in Japanese are a crossbreed of wild mallard and domestic ducks; they are free-ranging ducks in water-soaked rice paddy fields and are used for weed control and meat production. Domestic geese (*Anser cygnoides* var. *domestica*) are reared for food production on farms. We selected geese because wild geese (*A. indicus*) accounted for a large proportion of the deaths in AI (H5N1) outbreaks at Qinghai Lake in People’s Republic of China in 2005 (*5*). These 2 species of birds were obtained from the farm at 1 day of age and raised with commercial food in an isolated facility. Birds were moved into negative-pressure isolators of Biosafety Level 3–approved laboratories (National Institute of Animal Health, Tsukuba, Japan) for acclimation 1 week before inoculation.

Two different AI virus (H5N1) genotypes were used. A/chicken/Yamaguchi/7/2004 (Ck/Yama/7/04) is classified as genotype V (*13*). A/chicken/Miyazaki/K11/2007 (Ck/Miya/K11/07) belongs to genotype Z and H5 clade 2 subclade 2 (M. Mase, unpub. data), which is now circulating from China to Japan, Europe, and Africa (*5*,*14*). The stored virus was propagated for 36–48 hours in the allantoic cavity of 10-day-old embryonated chicken eggs at 37°C. The infectious allantoic fluid was harvested and stored at –80°C until use. All experimental procedures were approved by the Ethics Committee of the National Institute of Animal Health in Japan.

For each species, two 4-week-old birds were inoculated intranasally with 0.1 mL of the inoculum containing 10^8^ 50% egg infectious dose (EID_50_) per mL of each AI virus (H5N1). Each inoculated group was kept in a separate isolator. Inoculated birds were euthanized with an overdose injection of sodium pentobarbital (i.v.) on days 3 and 5 postinoculation.

For histopathology, the skin, including numerous feathers, was removed from the head, neck, back, shoulder, abdomen, thigh, and tail. Samples were fixed in 10% neutral-buffered formalin, embedded in paraffin, sectioned at 4 μm, and stained with hematoxylin and eosin. Immunohistochemistry was performed to detect the viral antigen with a Histofine Simple Stain PO (M) kit (Nichirei Inc., Tokyo, Japan). A mouse monoclonal antibody specific for the influenza A matrix protein (diluted 1:500; clone GA2B, AbD Serotec, Kidlington, UK) was used as the primary antibody (*11*). For the virus isolation, clean dry skin was collected from the neck and stored at –80°C (*11*). The viral titer of the samples was determined with 10-day-old embryonated chicken eggs and expressed as EID_50_/g as previously described (*13*). The viral titer <10^2^ EID_50_/g was considered negative for virus isolation. For the electron microscopic examination, flesh contour feathers were fixed in 3% glutaraldehyde in 0.1 M phosphate buffer, postfixed in 1% osmium tetroxide, and embedded in epoxy resin. Ultrathin sections were stained with uranyl acetate and lead citrate and examined under a Hitachi H-7500 transmission electron microscope (Hitachi Corp., Tokyo, Japan).

**Figure 1:**
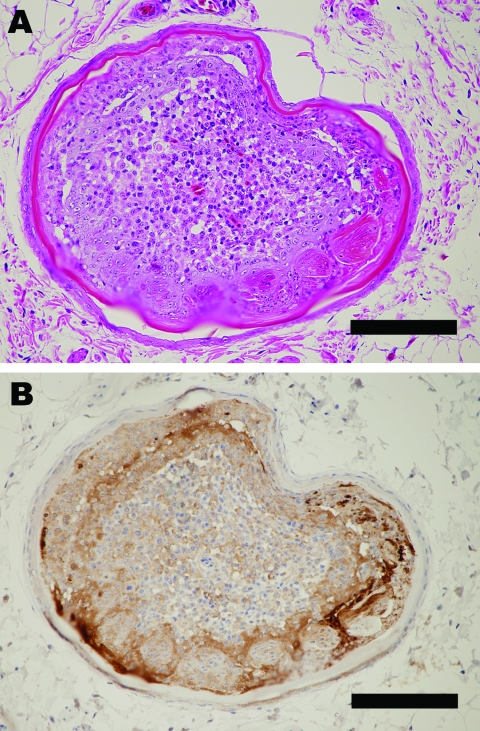
Pathologic changes in a goose infected with A/chicken/Miyazaki/K11/2007. A) Outer epidermal necrosis of the feather with inflammation in the inner feather pulp. Hematoxylin and eosin stain. Bar = 120 μm. B) Influenza viral antigens detected in feather epidermal cells. Immunohistochemistry. Bar = 120 μm.

**Figure 2:**
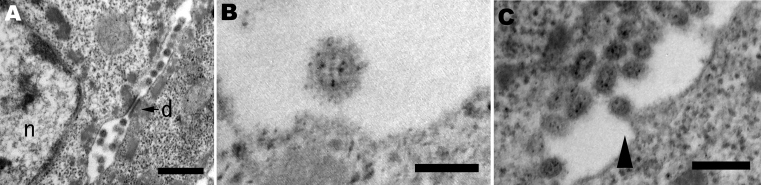
Pathology of a duck infected with A/chicken/Yamaguchi/7/2004. A) Electron microscopy of the feather epidermis showing virions observed between epidermal cells with the desmosome (d) and nucleus (n). Bar = 500 nm. B) Spherical virion with envelope spikes. Bar = 100 nm. C) Budding process of virion (arrowhead). Bar = 250 nm.

Inoculated birds did not exhibit apparent clinical signs, except for unilateral corneal opacity in a goose inoculated with Ck/Yama/7/04 on day 5 postinoculation. Results of histopathologic and virologic examinations are summarized in the Table. Histologically, viral antigens were occasionally detected in the feather epidermal cells with or without epidermal necrosis (Figure 1, panels **A** and **B**). Some affected feathers were accompanied by heterophilic and lymphocytic infiltration in the inner feather pulp. Other tissues in the skin were negative for influenza virus by immunohistochemical analysis with the exception of very rare positive reaction in stromal cells in the feather pulp. Virus isolation from the skin was positive in 1 duck and 1 goose inoculated with Ck/Yama/7/04; the viral titers were 10^3.5^ and 10^4.5^ EID_50_/g, respectively. All ducks and geese inoculated with Ck/Miya/K11/07 tested positive for the isolation; the viral titers were 10^2.5^–10^4.5^ EID_50_/g. Ultrastructurally, round, enveloped virions 80 to 100 nm in diameter were observed between feather epidermal cells in both domestic ducks and geese (Figure 2, panels **A** and B). Spherical virions budding from cell surface were occasionally observed (Figure 2, panel **C**).

**Table:** Histopathology of feathers and virus isolation from the skin in domestic ducks and geese inoculated with 2 different avian influenza (H5N1) genotypes*

PID	A/chicken/Yamaguchi/7/2004			A/chicken/Miyazaki/K11/2007	
Duck	Goose	Duck	Goose
3	+/+† (3.5)‡	–/– (–)		+/+ (4.5)	+/+ (4.4)
5	+/+ (–)	+/+ (4.5)		–/+ (3.8)	+/+ (2.5)

*PID, postinoculation day. †Epidermal necrosis of feathers/detected viral antigens; +, positive; –, negative. ‡Viral titer of the skin expressed as log_10_ egg infectious dose (EID_50_)/g; –, negative (<10^2^ EID_50_/g).

## Conclusions

We found that 2 different AI virus (H5N1) genotypes that were isolated in 2004 and 2007 can replicate in the feather epidermal cells of domestic ducks and geese. To our knowledge, this is the first report of in vivo ultrastructural observation of AI (H5N1) replication in waterfowl.

The important finding is that the histologic feather finding and virus isolation from the skin were found in inoculated birds that did not exhibit apparent clinical signs. Although 1 goose inoculated with Ck/Yama/7/04 was negative for all examinations, this might have resulted from individual differences in susceptibility or the limited area of the skin used for the examination. Nevertheless, our data indicate that recent AI (H5N1) strains are likely to replicate in feather epidermal cells of domestic ducks and geese. All birds inoculated with Ck/Miya/K11/07, which belongs to the current lineage spreading to Europe and Africa, tested positive for virus isolation, compared with the results with Ck/Yama/7/04. Feathers can easily drop off, blow away, or be reduced to dust, suggesting that affected feathers of waterfowl infected with influenza (H5N1) virus can be potential sources of infection, along with their feces and respiratory secretions (*7**,**8*). At this time, it is unclear to what extent affected feathers contribute to the epidemiology of AI (H5N1) field outbreaks. However, more attention needs to be paid to persons who handle domestic waterfowl possibly infected with AI virus (H5N1).
